# Targeting liver angiotensinogen with siRNA enhances podocyte regeneration through renin lineage cells in experimental kidney disease

**DOI:** 10.1016/j.omtn.2026.103065

**Published:** 2026-08-19

**Authors:** Loïs A.K. van der Pluijm, Angela Koudijs, A.H. Jan Danser, Joris I. Rotmans, Roel Bijkerk

**Affiliations:** 1 Department of Internal Medicine (Nephrology) and the Einthoven Laboratory for Vascular and Regenerative Medicine, Leiden University Medical Centre, 2333ZA Leiden, the Netherlands; 2 Division of Pharmacology and Vascular Medicine, Department of Internal Medicine, Erasmus MC, 3015GD Rotterdam, the Netherlands

**Keywords:** MT: oligonucleotides, therapies and applications, angiotensinogen, small interfering RNA, renin-angiotensin system, cell of renin lineage, chronic kidney disease, podocyte, plasticity, adult progenitor

## Abstract

Targeting angiotensinogen (AGT) with small interfering RNAs (siRNAs) is a promising strategy to suppress renin-angiotensin system (RAS) activity and to potentially mitigate progression of chronic kidney disease (CKD). Although AGT siRNAs have been shown to exert kidney-protective effects in experimental models of kidney injury, the underlying mechanisms remain unclear. Juxtaglomerular cells of renin lineage (CoRL) have recently been recognized as progenitors capable of replenishing several glomerular cell types during injury. Here, we investigated whether AGT siRNA alters CoRL-driven glomerular cell repopulation in the 5/6 nephrectomy (5/6NX) model using renin lineage-tracing mice treated with either AGT siRNA or luciferase-targeting control. AGT silencing achieved ∼85% reduction in plasma AGT and induced a marked compensatory rise in plasma renin concentration. Despite these systemic effects, AGT knockdown did not alter kidney function, as reflected by unaffected blood urea levels, urinary albumin-to-creatinine ratios, glomerular hypertrophy, or kidney fibrosis. While the number of CoRL-derived mesangial cells remained unchanged, AGT inhibition resulted in a modest but significant increase in CoRL-derived parietal epithelial cells and a pronounced increase in CoRL-derived podocytes. These findings indicate that liver-directed AGT silencing does not confer short-term functional benefit but enhances CoRL-mediated podocyte regeneration, identifying a potential mechanism that may contribute to delayed renal repair.

## Graphical abstract

**Figure:**
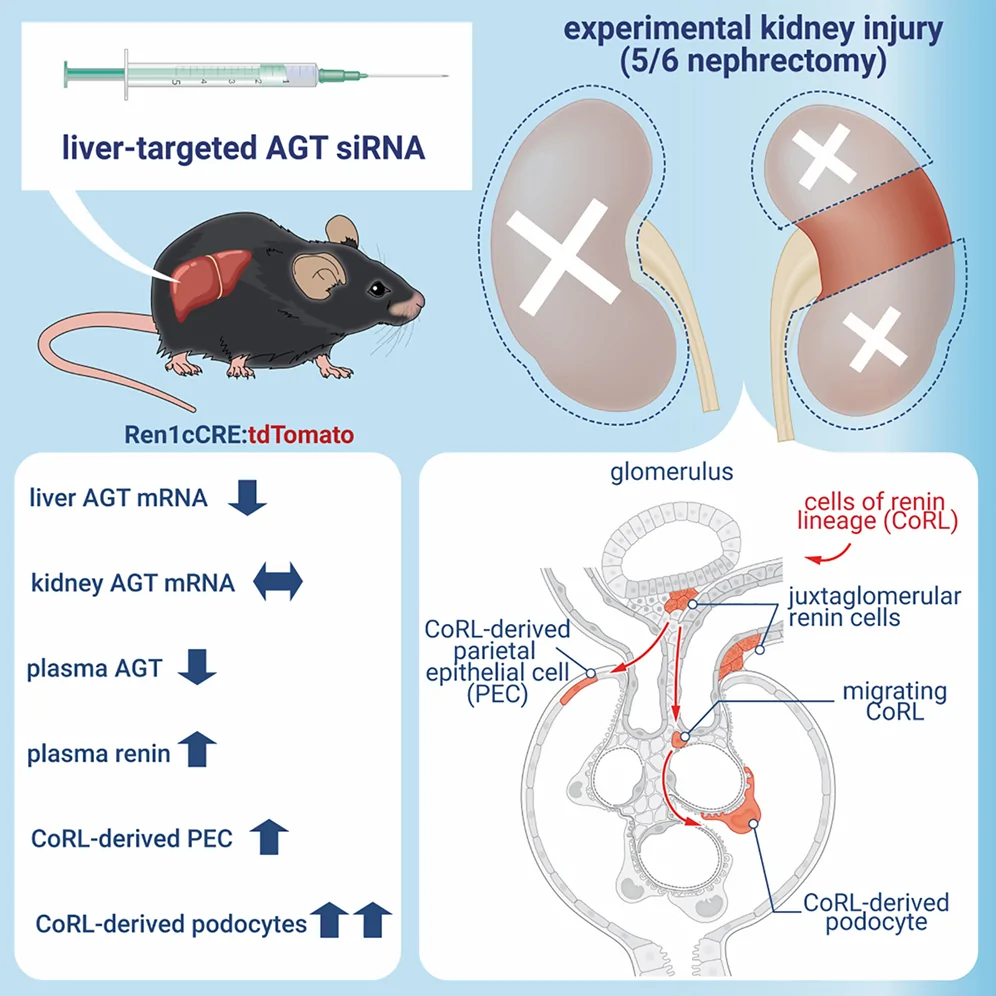

## Teaser

Cells of renin lineage possess regenerative potential following kidney injury. Liver-directed AGT siRNA, a novel RNA-based approach to inhibit the renin-angiotensin system, enhances their differentiation toward glomerular epithelial cell fates, revealing a potential mechanism underlying its long-term renoprotective effects.

## Introduction

Chronic kidney disease (CKD) affects approximately 10% of the global population and is a major contributor to cardiovascular mortality.^1^ With the global rise of non-communicable diseases such as diabetes, obesity, and hypertension, CKD is projected to become the fifth leading cause of death by 2040.^1^ Current CKD management commonly includes renin-angiotensin system (RAS) blockade to control hypertension and protect kidney function.

A novel approach to RAS modulation involves small interfering RNAs (siRNAs) targeting angiotensinogen (AGT).^2^ By suppressing hepatic AGT synthesis, a single injection of AGT-directed siRNA can achieve long-lasting RAS inhibition, lasting up to six months, without major adverse effects.^3^ This strategy has the potential to overcome antihypertensive therapy non-adherence, a key barrier to adequate blood pressure control. Preclinical studies demonstrate that AGT siRNA lowers blood pressure and mitigates end-organ damage to a similar extend as classical RAS blockers, while phase 1 and 2 clinical trials confirm its sustained antihypertensive efficacy.^3^

Renoprotective effects of AGT siRNA have been demonstrated in the 5/6 nephrectomy model as well as in experimental diabetes using transgenic TGR(mRen2)27 rats.^4^^,^^5^ Notably, in both settings, AGT siRNA improved kidney outcomes independently of blood pressure changes, likely through reduced renal angiotensin II generation from liver-produced AGT.^4^^,^^5^ However, the full spectrum of mechanisms underlying these effects remains unclear.

Intriguingly, RAS inhibition has been shown to influence the regenerative capacity of cells of renin lineage (CoRL).^6^ Traditionally recognized as renin-producing cells within the juxtaglomerular apparatus,^7^ CoRL have recently emerged as an intrinsic progenitor population within the kidney.^8^^,^^9^^,^^10^ Following injury, such as experimental focal segmental glomerulosclerosis (FSGS) or 5/6 nephrectomy, CoRL can replenish podocytes, mesangial cells, and parietal epithelial cells (PECs) in the glomerular tuft and Bowman’s capsule.^8^^,^^9^^,^^10^ Moreover, subsets of perivascular CoRL including erythropoietin-producing cells,^11^ act as interstitial pericytes that may differentiate into myofibroblasts during fibrosis.^12^^,^^13^

Classical RAS blockade with enalapril (angiotensin-converting enzyme (ACE) inhibition) or losartan (AT1R blockade) has been shown to enhance CoRL proliferation, promote their migration into the glomerulus, and stimulate transdifferentiation toward podocytes, PECs, and mesangial cells in experimental FSGS.^6^

Here, we sought to determine whether liver-targeted AGT siRNA similarly modulates CoRL-mediated regeneration. Such an effect could represent a previously unrecognized mechanism contributing to the renoprotective actions of AGT siRNA.

## Results

### siRNA-mediated liver-specific AGT knockdown reduces circulating AGT and increases PRC

**Figure 1:**
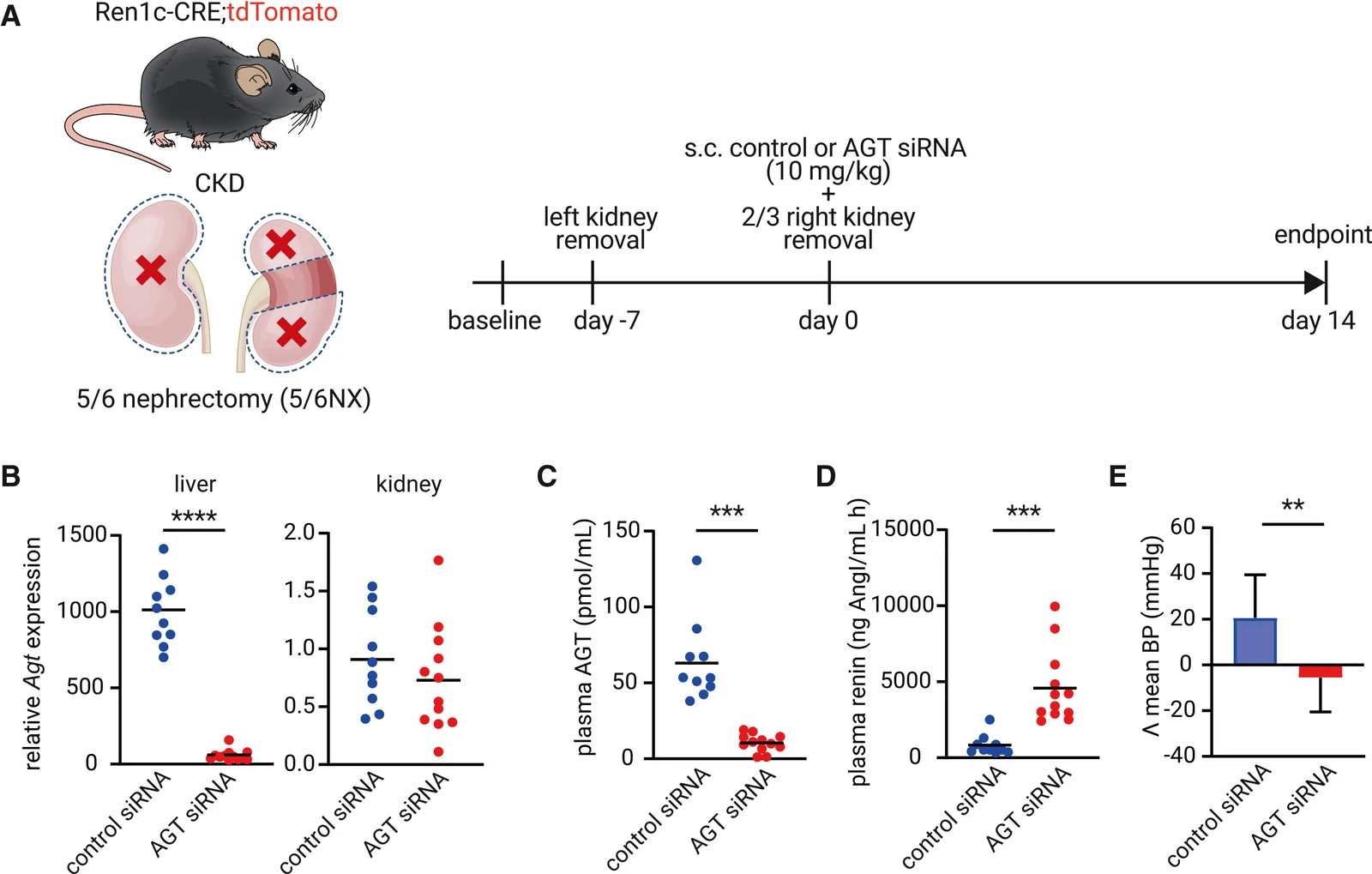
Experimental setup and systemic effects of AGT siRNA treatment in 5/6 nephrectomized Ren1c-CRE;tdTomato mice (A) Schematic overview of the experimental design. Ren1c-CRE;tdTomato mice underwent 5/6 nephrectomy (5/6NX) and received control or AGT siRNA according to the indicated timeline. (B) Relative *Agt* mRNA expression (normalized to GAPDH) in liver and kidney tissue at the experimental endpoint. (C) Plasma AGT levels at the experimental endpoint, demonstrating effective systemic AGT reduction following AGT siRNA treatment. (D) Plasma renin concentration at the experimental endpoint. (E) Change in mean blood pressure from baseline to the experimental endpoint. Data are presented as mean ± SD; each dot represents one animal (*n* = 11–12 per group). ∗∗*p* < 0.01, ∗∗∗*p* < 0.001, ∗∗∗∗*p* < 0.0001.

To assess the impact of AGT inhibition on the regenerative capacity of cells of the renin lineage (CoRL), we used Ren1c-Cre;tdTomato mice, which enable lineage tracing of CoRL. Mice received either AGT-targeting siRNA or control luciferase siRNA and were subjected to 5/6 nephrectomy (5/6NX), followed by a 14-days monitoring period (Figure 1A). Efficient liver-specific target engagement was confirmed by a ∼95% reduction in hepatic *Agt* mRNA expression, whereas renal *Agt* expression remained unchanged (Figure 1B). Hepatic AGT knockdown resulted in a marked reduction in circulating AGT levels (62.33 ± 26.33 vs 10.47 ± 5.70 pmol/mL), corresponding to an ∼85% decrease (Figure 1C). As expected, this reduction was accompanied by a pronounced compensatory increase in plasma renin concentration (PRC; 805.4 ± 630.9 vs 4586 ± 2436 ng AngI/mL/h; Figure 1D). In line with the elevated PRC, AGT siRNA-treated mice also exhibited a significantly higher juxtaglomerular index, reflecting a greater proportion of glomeruli with renin-positive staining at the juxtaglomerular apparatus (Figure S1). AGT siRNA treatment also attenuated 5/6NX-induced hypertension (Figure 1E).

### No apparent effect of AGT siRNA on kidney function or morphology

**Figure 2:**
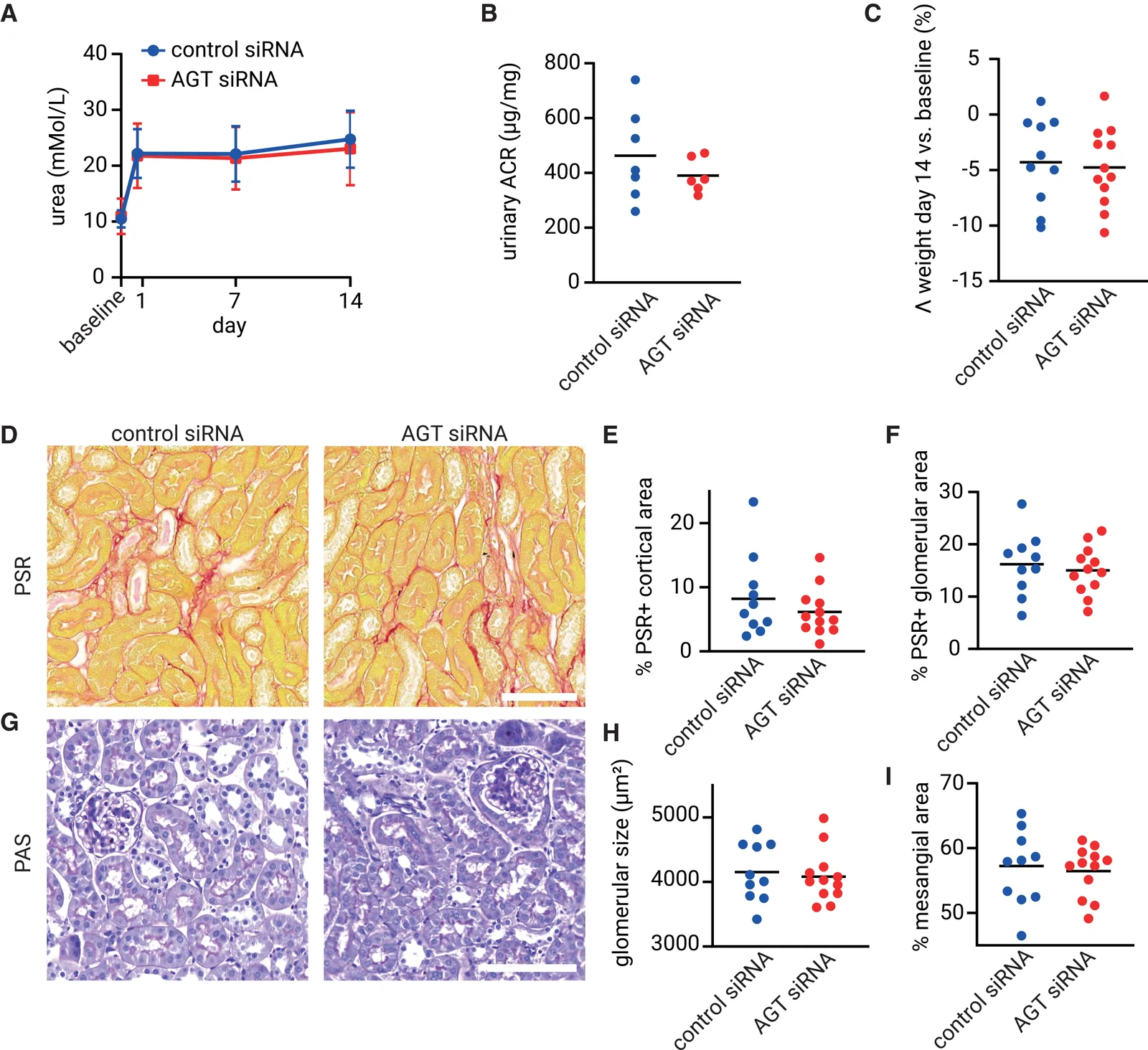
Renal function and histological assessment following AGT siRNA treatment (A) Blood urea levels measured throughout the study show no significant difference in kidney function with or without AGT siRNA treatment. (B) Urinary albumin-to-creatinine ratio (ACR) at endpoint. (C) Percentage change in body weight (endpoint vs. baseline). (D–F) Representative Picro Sirius Red (PSR) staining and corresponding quantification reveal comparable levels of kidney fibrosis between groups. (G–I) Periodic acid-Shiff (PAS) staining with quantification of glomerular area and percentage mesangial expansion. Scale bars, 100 μm. Data are presented as mean (±SD); each dot represents 1 animal (*n* = 11–12 per group).

We next evaluated the effect of siRNA-mediated AGT knockdown on kidney function. As expected, blood urea levels increased following 56NX; however, AGT siRNA treatment did not alter urea concentrations compared to controls (Figure 2A). Urinary albumin-to-creatinine ratios remained similar between groups upon AGT knockdown (Figure 2B) and both groups showed a comparable reduction in body weight over the 14-days study period (Figure 2C).

To assess potential structural effects associated with AGT knockdown, we examined fibrotic remodeling and glomerular morphology. Picro Sirius Red (PSR) staining revealed no difference in collagen deposition between groups (Figures 2D–2F). Likewise, AGT inhibition did not affect glomerular area (Figures 2G and 2H) or mesangial expansion (Figures 2G and 2I), indicating no detectable impact on glomerular hypertrophy of matrix accumulation.

### Inhibiting AGT increases CoRL-derived podocytes after 5/6NX

In our previous work, we demonstrated that podocytes, mesangial cells, and PECs can be replenished by CoRL following experimental kidney injury.^14^ Here, we examined whether AGT siRNA treatment affects glomerular CoRL abundance and differentiation following 5/6 nephrectomy.

**Figure 3:**
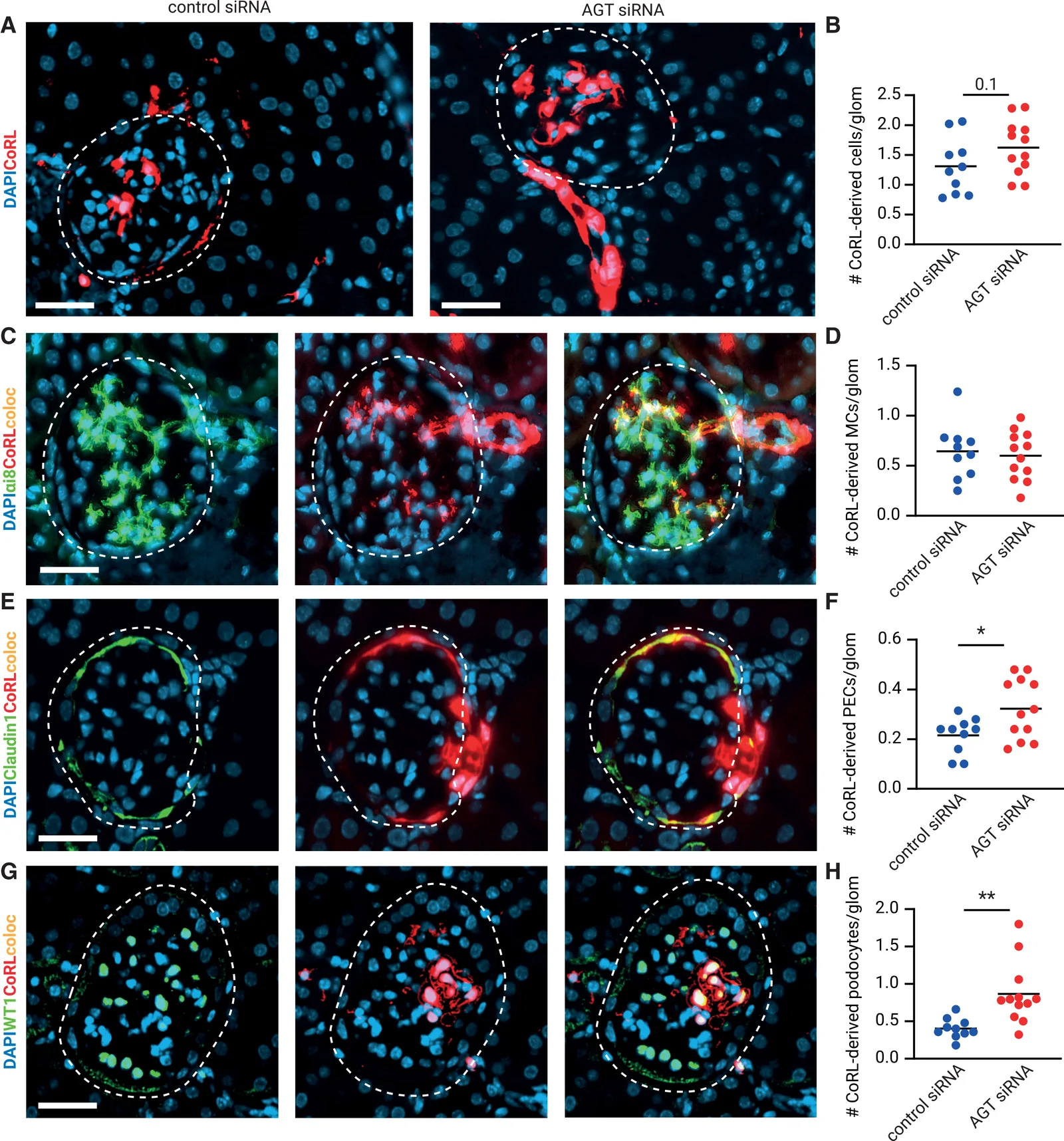
Contribution of cells of renin lineage to different glomerular cell types upon AGT siRNA treatment (A) Representative fluorescence images of glomeruli showing tdTomato+ cells (cells of renin lineage, CoRL). (B) Quantification of tdTomato+ cells per glomerulus following AGT siRNA treatment. (C and D) Co-immunofluorescence staining for WT1 (podocytes) and tdTomato, with overlay and quantification of double-positive cells per glomerulus. (E and F) Co-staining for Claudin-1 (parietal epithelial cells, PECs) and tdTomato, with corresponding quantification. (G and H) Co-staining for α8-integrin (mesangial cells) and tdTomato, with quantification. Dashed lines indicate glomeruli. Scale bars, 50 μm. Data are presented as mean; each dot represents 1 animal (*n* = 10–12 per group). ∗*p* < 0.05, ∗∗*p* < 0.01

Histological analysis of endogenous tdTomato fluorescence (marking CoRL lineage) within glomeruli revealed a non-significant trend toward increased numbers of glomerular tdTomato+ cells following AGT knockdown (1.310 ± 0.470 cells vs 1.622 ± 0.453 cells per glomerulus from CoRL origin, *p* = 0.1, Figures 3A and 3B).

To assess the fate of these CoRL-derived cells, kidney sections were co-stained for tdTomato together with markers for podocytes (WT1), mesangial cells (integrin α8), and PECs (claudin-1). The number of CoRL-derived mesangial cells was unchanged upon AGT siRNA treatment (Figures 3C and 3D). In contrast, AGT knockdown led to a modest, but significant increase in CoRL-derived PECs (0.215 ± 0.073 vs 0.322 ± 0.121 cells per glomerulus from CoRL origin, *p* < 0.05; Figures 3E and 3F) and a pronounced increase in CoRL-derived podocytes (0.404 ± 0.133 vs 0.867 ± 0.415 cells per glomerulus from CoRL origin, *p* < 0.01; Figures 3G and 3H). Additional podocin (NPHS2) immunostaining demonstrated expression of the slit diaphragm protein podocin in CoRL-derived glomerular cells (Figure S2), further supporting differentiation toward a mature podocyte phenotype. In contrast to the glomerular effects, AGT siRNA did not alter the abundance of α-smooth muscle actin (αSMA)-positive CoRL-derived cells in the renal interstitium (Figure S3).These data suggest that AGT siRNA enhances the regenerative contribution of CoRL to glomerular epithelial cell fates following 5/6NX.

## Discussion

This study expands current understanding of liver-targeted AGT siRNA by confirming its efficacy in experimental kidney disease by reducing circulating AGT and preventing the 5/6NX-induced rise in blood pressure. While no direct short-term renoprotective effect was observed, AGT siRNA markedly increased the number of CoRL-derived podocytes within glomeruli. This finding aligns with previous studies demonstrating CoRL-derived glomerular cell turnover within two weeks after 5/6NX,^14^ indicating that this is a biologically relevant time window for early regenerative responses. However, this relatively short time frame may also explain the absence of measurable functional improvement.

Indeed, earlier work in the 5/6NX rat model for CKD, known to respond to RAS blockade,^15^ showed that two weeks of AGT siRNA treatment did not yet improve kidney function, and in some settings even worsened it under low-salt conditions.^16^ In contrast, reduced proteinuria was observed after four weeks of treatment.^4^ Together, these findings suggest that the renoprotective effects of AGT siRNA may become more apparent over time. While no functional improvement was observed at the 14-days endpoint in the present study, longer term studies are required to determine whether similar delayed benefits occur in this model.

Importantly, we found that AGT siRNA stimulated the differentiation into PECs, and more prominently, into podocytes. This is in line with previous studies showing that classical RAS inhibition via ACE or AT1R blockade promotes similar regenerative processes.^6^ Our findings extend previous observations of injury-induced CoRL plasticity, demonstrating that AGT silencing further enhances this regenerative response.^14^ No differences were observed in interstitial αSMA-positive CoRL-derived cells, suggesting that these effects were largely confined to glomerular CoRL fates. The concurrent increase in PECs and podocytes raises the intriguing possibility that CoRL-derived PECs may serve as intermediate progenitors in podocyte regeneration, as previously proposed by others.^17^ Future studies will be required to elucidate this lineage relationship. It is conceivable that the progressive accumulation of CoRL-derived podocytes over time contributes to delayed renal repair, representing a potential additional mechanism of AGT siRNA-mediated benefit. Whether these cellular changes ultimately translate into sustained renoprotection remains to be established.

Beyond these potentially beneficial effects, recent reports have noted that RAS blockade can also induce concentric arteriolar hypertrophy involving CoRL.^18^^,^^19^ Comparable vascular changes have been described following AGT siRNA administration,^20^ emphasizing the importance of monitoring CoRL fate under different renoprotective interventions.

Taken together, our study illustrates that AGT siRNA effectively reduces circulating AGT and blood pressure in murine experimental kidney injury, while promoting CoRL-derived podocyte and PEC formation. Although no short-term improvement in kidney function was detected, the observed cellular responses may underlie delayed renoprotective effects with prolonged treatment. These findings identify a potential novel mechanism of AGT siRNA-mediated kidney protection and further support its therapeutic promise in CKD.

## Materials and methods

### Animals and surgical procedures

Male C57BL/6J Ren1-Cre^21^ × Rs-tdTomato-R mice (9–12 weeks) were used in all experiments (*n* = 11–12 per group). Animals were maintained under standard housing conditions (20°C–22°C, 55% humidity, 12 h light/dark cycle) with free access to food and water. All experiments complied with Dutch legislation (Experiments on Animals Act, 2014 revision) and EU Directive 2010/63/EU, and were approved by the Animal Ethics Committee of Leiden University Medical Center, the Netherlands (permit no. AVD1160020171145).

The 5/6 nephrectomy (5/6NX) model was performed as previously described.^13^^,^^14^ In brief, mice underwent unilateral nephrectomy followed by partial contralateral nephrectomy one week later. Fourteen days after the second surgery, animals were euthanized by exsanguination under isoflurane anesthesia with peri- and postoperative analgesia (buprenorphine, 0.1 mg/kg subcutaneously, s.c.). Seven mice did not reach the experimental endpoint following 5/6 nephrectomy (4 control siRNA and 3 AGT siRNA) and were excluded from all analyses, resulting in final group sizes of *n* = 10 (control siRNA) and *n* = 12 (AGT siRNA).

### Treatment with AGT siRNA

On the morning prior to the second 5/6NX surgery, mice received an s.c. injection of 10 mg/kg chemically modified GalNAc-conjugated AGT-targeting siRNA or control luciferase-targeting siRNA (2.5 mg/mL in PBS; Alnylam Pharmaceuticals). GalNAc conjugation enables selective uptake by hepatocytes via the asialoglycoprotein receptor.^4^

### Blood urea, uACR

Blood urea was measured in tail vein samples using a Reflotron Plus System (Roche Diagnostics). Urine was collected at sacrifice from animals with urine present in the bladder and analyzed using ELISA kits for albumin (80630, Crystal Chem) and creatinine (80350, Crystal Chem) to calculate urinary albumin-to-creatinine ratio (uACR).

### Blood pressure measurement

Systolic and diastolic blood pressure was measured in a subset of mice before and after siRNA treatment using a non-invasive tail cuff system (CODA, Kent Scientific). Each session consisted of 10 acclimatization cycles followed by 10 measurement cycles. The mean of all valid systolic and diastolic readings was calculated to determine average blood pressure for each animal.

### Plasma AGT and renin measurements

Plasma was obtained by centrifugation of whole blood collected in K2 EDTA-buffered tubes. Plasma AGT and renin concentrations were determined as previously described.^4^ In brief, AGT was quantified using an enzyme kinetic assay measuring the maximal amount of angiotensin I (Ang I) generated during incubation with rat-derived renin in the presence of ACE, angiotensinase, and serine protease inhibitors. Active PRC was determined by quantifying Ang I generation in the presence of excess of porcine-derived AGT.

### Histology and immunohistochemistry evaluation

Kidneys were fixed in 10% neutral-buffered formalin for 2 h, transferred to 70% ethanol overnight, and paraffin embedded. Sections (4 μm) were stained with periodic acid-Shiff (PAS) reagent (Sigma) for 45 min and counterstained with hematoxylin for 30 s. Mesangial area was quantified in 50 glomeruli per section using the isodata auto-threshold algorithm on 16-bit images.^22^ Collagen I and III were visualized by an 1 h incubation in 0.1% PSR solution, followed by 2 rinses in 1% acidic acid. PSR staining was quantified over the entire kidney section using HistoQuant software (3DHISTECH).

For immunofluorescence, sections underwent heat-induced antigen retrieval and blocking, followed by overnight incubation at 4°C with primary antibodies against renin (1:200, 50279-R001, SinoBiological), tdTomato (1:500, AB1140-Origene, or 1:500, LS-C60076-Isbio), WT1 (1:500, AB89901-Abcam), NPHS2 (1:1,000, AB50339-Abcam), integrin α8 (1:200, AF4076, R&D), claudin 1 (1:200, RB-9209, Thermo Fisher Scientific), and αSMA (1:200, mab1420, R&D). Alexa Fluor-conjugated donkey secondary antibodies (1:250) were applied for 2 h at room temperature. Renin immunostaining was quantified using the juxtaglomerular index, calculated as the number of glomeruli with renin-positive staining at the juxtaglomerular apparatus divided by the total number of glomeruli (≥50) assessed and expressed as a percentage. Glomerular colocalization was assessed by manual counting in ≥50 glomeruli per section per animal. Interstitial colocalization was analyzed using QuPath with fixed intensity thresholds applied uniformly across all images.

### Statistics

Data were analyzed using GraphPad Prism. A *p* value < 0.05 was considered statistically significant. Urea levels over time were analyzed using two-way repeated measures ANOVA with Tukey post hoc testing. All other comparisons between two groups were performed using unpaired Student’s *t* tests.

## Data and code availability

Data supporting the findings of this study are available from the corresponding author upon reasonable request.

## Acknowledgments

The authors kindly acknowledge funding from the 10.13039/501100002997Dutch Kidney Foundation (20OK015) and EFSD/Novo Nordisk Foundation Future Leaders Award Program (NNF23SA0087433). AGT siRNA was kindly provided by 10.13039/100006400Alnylam Pharmaceuticals. The mouse strain in this study was created with the support of the Roswell Park Comprehensive Cancer Center and 10.13039/100000054National Cancer Institute (NCI) grant P30CAO16056 and was kindly provided by Professor Kenneth W. Gross. Illustrations used in the graphical abstract were created by Manon Zuurmond.

## Author contributions

L.A.K.v.d.P., conceptualization, investigation, writing – original draft, methodology, visualization, writing – review and editing, and formal analysis; A.K., investigation, methodology, and writing – review and editing; A.H.J.D., investigation and writing – review and editing; J.I.R., writing – review and editing; R.B., conceptualization, funding acquisition, writing – original draft, methodology, writing – review and editing, visualization, project administration, and supervision.

## Declaration of interests

The authors declare no competing interests.

## Supplemental information

Document S1. Figures S1–S3

Document S2. Article plus supplemental information
