# Supplementary material for: Targeting liver angiotensinogen with siRNA enhances podocyte regeneration through renin lineage cells in experimental kidney disease
Source: Mol Ther Nucleic Acids. 2026 Aug 19;37(3):103065. doi: 10.1016/j.omtn.2026.103065 (PMC13559801; doi:10.1016/j.omtn.2026.103065)
Supplement: Supplementary file 1 [file mmc1.pdf]

OMTN, Volume *37*

Supplemental information

Targeting liver angiotensinogen with siRNA enhances podocyte regeneration through renin lineage cells in experimental kidney disease

Loïs A.K. van der Pluijm, Angela Koudijs, A.H. Jan Danser, Joris I. Rotmans, and Roel Bijkerk

Supplemental material

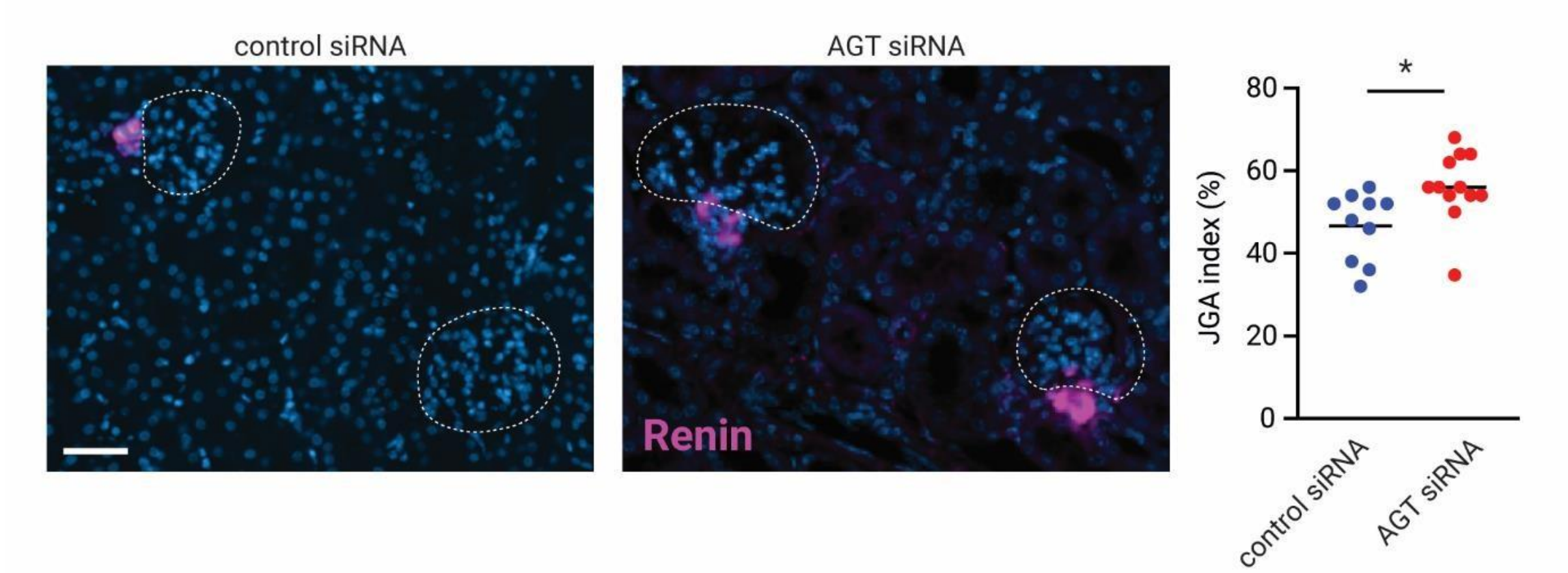

Figure S1. Renin expression following AGT siRNA treatment in 5/6 nephrectomized mice. Representative images of renal renin immunostaining in control siRNA- and AGT siRNA-treated mice. The juxtaglomerular index was calculated as the percentage of glomeruli with renin-positive staining at the juxtaglomerular apparatus. Dashed lines indicate glomeruli. Scale bar = 50 µm Data are presented as mean. *P<0.05

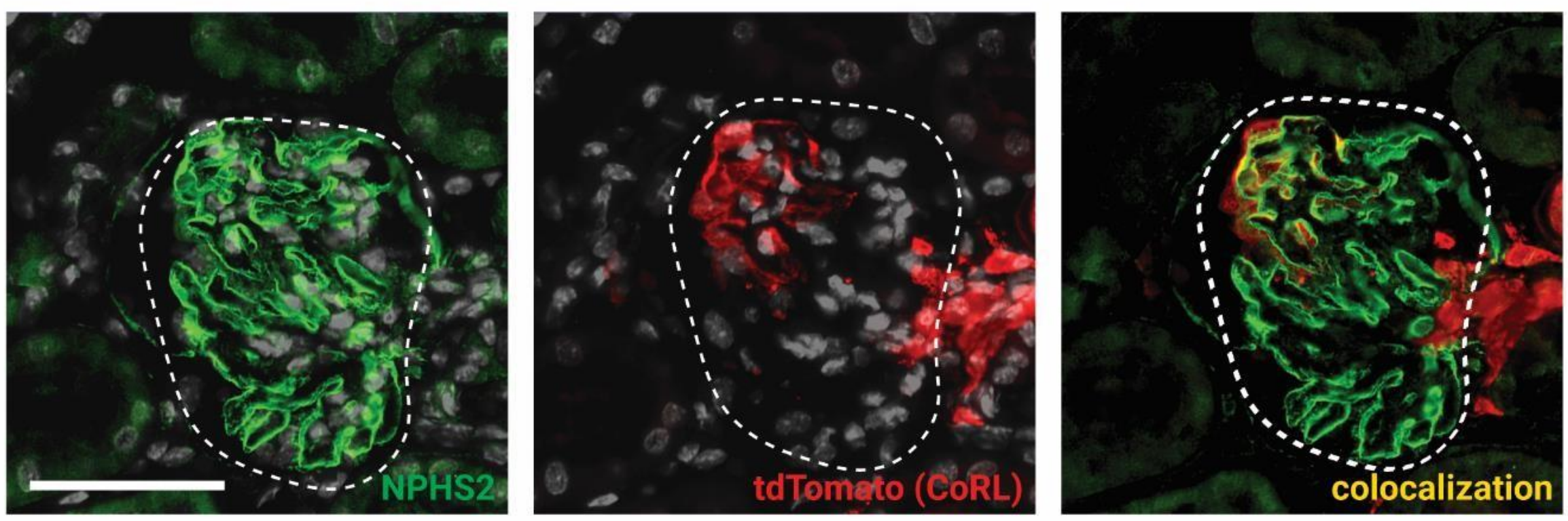

Figure S2. Podocin (NPHS2) expression in CoRL-derived glomerular cells following AGT siRNA treatment. Representative immunofluorescence images showing podocin (NPHS2, green), endogenous tdTomato fluorescence marking cells of the renin lineage (CoRL; red), and the merged image demonstrating colocalization within the glomerulus. The dashed line outlines the glomerulus. Scale bar = 50 µm.

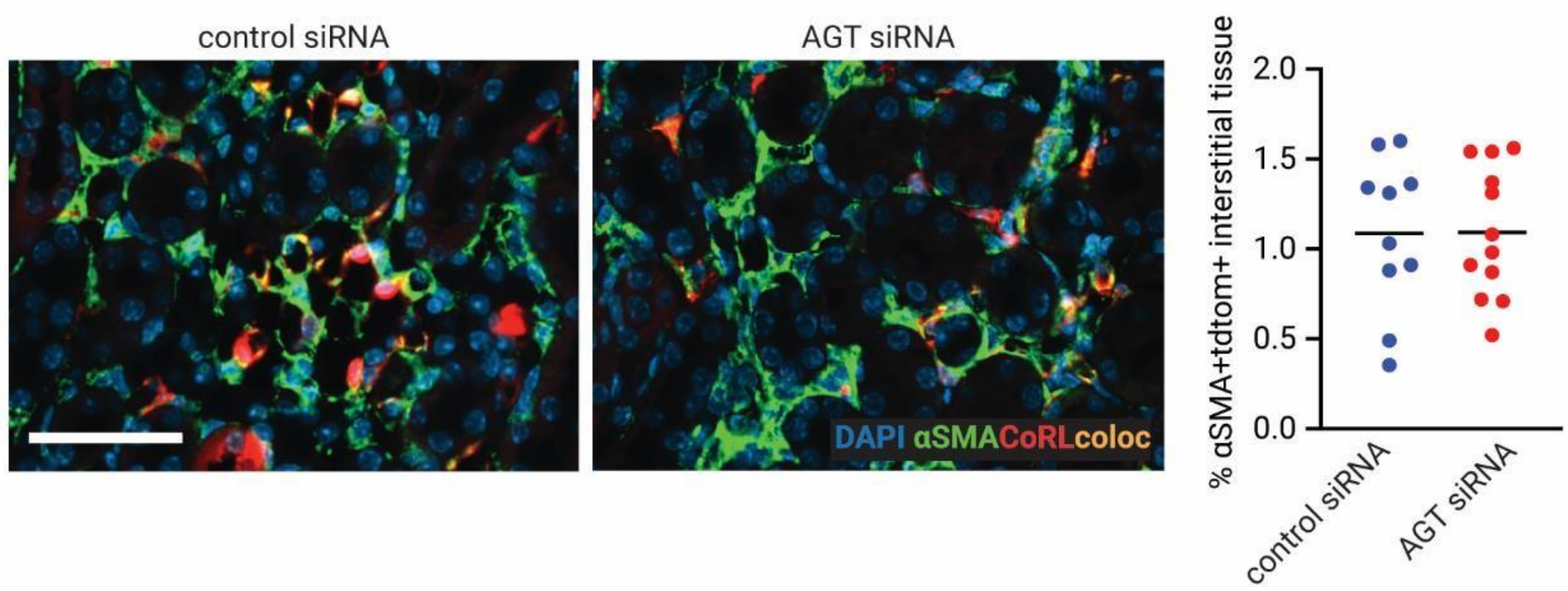

Figure S3. AGT siRNA does not alter interstitial CoRL-derived αSMA-positive cells after 5/6 nephrectomy. Representative immunofluorescence images of kidney cortex sections stained for tdTomato (red) and αSMA (green) in control siRNA- and AGT siRNA-treated mice. Scale bar = 50 µm. Data is presented as mean.
